# Sexual coercion at sexual debut and associated factors among young females in rural areas of Boset District, Eastern Ethiopia: a mixed method study

**DOI:** 10.4314/ahs.v22i3.4

**Published:** 2022-09

**Authors:** Sileshi Garoma Abeya

**Affiliations:** Department of Public Health, Adama Hospital Medical College, Adama, Ethiopia

**Keywords:** Coercion, sexual debut, rural, Ethiopia

## Abstract

**Objectives:**

Assess the prevalence and associated factors of sexual coercion at sexual debut among young females in rural areas of Boset district, Oromia Region, Eastern Ethiopia.

**Methods:**

Cross-sectional community based study design was conducted using both qualitative and quantitative data collection methods. A sample of 632 young females aged 10–24 years was taken from six rural Kebeles by systematic random sampling for quantitative and data were collected using a pre-tested structured questionnaire. The collected data was analyzed using SPSS version 23. Principally Binary Logistic regression model was fitted. Moreover, eight focus group discussions targeting different groups were held andanalyzed thematically.

**Results:**

The prevalence of sexual coercion at sexual debut was 36.5% (95%CI: 34.6%–38.4%) among sexually active respondents. Married young female (AOR, 0.71; 95%CI: 0.42, 0.81), living away from their parents (AOR, 5.07; 95%CI: 1.07,24.01), age group of 15- 19 years (AOR, 0.19; 95%CI: 0. 06, 0.54), alcohol consumption (AOR, 2.44; 95%CI: 1.17, 5.08) and Khat chewing (AOR, 8.30; 95%CI: 1.89, 36.38) were factors predicting the likelihood of having sexual coercion at sexual debut.

**Conclusion:**

Sexual coercion at sexual debut is a major public health problem among young females in the study community. The emerging program interventions need to take this problem into consideration.

## Background

Sexual coercion is the act of being physically, psychologically, financially or otherwise forced or tricked into engaging in sexual activity^1^, ^2^. Most commonly the victims of sexual coercion are women and children^3^, ^4^. These includes acts of being worn down by someone who repeatedly asks for sex being lied to or being promised things that weren't true to trickou into having sex; having someone threaten to end a relationship or spread rumors about you if you don't have sex with them; and having an authority figure, like a boss, property manager use their influence or authority to pressure young female into having sex^5^, ^6^. It is commonly believed that sexual coercion is perpetrated by the male sex against the female sex^5^. For example, in a community based study 33% of young peoples reported having sexual coercion and 70% of them new their perpetrator (a boyfriend and an acquaintance)^2^. Studies in the fields of sexual coercion at sexual debut, including rape are usually based on biased estimates to elicit the relationship between the victims and rapist characterization of the prevalence is challenging^2^, ^7^. There are also popular myths among both victims and perpetrators^8^. Some of the commonly perceived myths are sexual coercion is performed by strangers in dangerous places; the offender or perpetrators are deviates or distorts, and they are more likely to socially and psychologically normal acquaintances^2^, ^8^.

Study examined sexual coercion at first sexual debut among rural young females in low-to-middle income counties found 23% on average^9^, ^10^. Modifiable predictors of sexual coercion at firs sexual debut are poorly understood, particularly among young people in rural areas who may have less access to sexual health resources^6^, ^7^, ^11^. In the context of sexual coercion drugs/alcohol consumption by both men and women is one of the most frequently cited to lower the victims inhibition (loosen them up) or lesson their verbal resistance of sexual advances. Ages, parental communication, marital status, education, living arrangements are among many factors frequently associated with sexual coercion at sexual debut^4^, ^12^–^14^.

Sexual coercion has been linked to a variety of negative outcomes such as psychiatric symptoms, poor school performance, and behavioral problems^4^, ^15^; among adolescent girls, it has been associated with pregnancy and abortion^11^. Sexual coercion also may increase adolescents' risk for infection with human immunodeficiency virus (HIV) and other sexually transmitted infection^6^.

However, there is a paucity of information on the prevalence and associated factors estimates of sexual coercion at first sexual debut among young people in urban and university settings of Ethiopia^12^, ^16^–^19^. However, in rural Ethiopia, where more than 80% of the young people live, the prevalence and associated factors of sexual coercion at first sexual debut is not well explored. Obtaining reliable estimates of sexual coercion among rural adolescents is important given the challenging social norms and myths. The study assesses both prevalence and associated factors of sexual coercion at first sexual debut and associated factors in the rural settings of Ethiopia during 2019.

## Methods

### Study Area

The study was conducted in rural Kebeles (the lowest administrative unit in government structure) among young females (10- 24 years) in Boset district, Eastern Ethiopia. The district is found in East Shoa Zone of Oromia Region 120 kilometers away from Addis Ababa. The district has a total population of 497, 143 during in 2019/20 projected from 2007 Census conducted by the Central Statistical Agency of Ethiopia, considering 2.9 % as rate of natural increase^20^. The total number of those aged 10–24 years in the district was estimated to be 154, 114 based on the assumption of 31% of all segment of the population^20^.

### Study Design and Period

A community based cross-sectional design was employed using both quantitative and qualitative data collection methods from May to June, 2019.

### Population

All young females (10–24 years) and those living in randomly selected households of rural Kebeles in Boset district were the source and study population, respectively. Those young females who have lived at least for six months in the district prior to the survey were included, while those critically ill and unable to respond to the questionnaire were excluded from the study.

### Sample Size

The required sample size was calculated by a single population proportion formula using 50% proportion of sexual coercion among young females of in the absence the absence of information from rural settings, at 95% confidence level, precision of 5%, design effect of 1.5 and adding 15 percent for the possible non responses due to the sensitive nature of the study. Finally, a minimum of 662 study subjects was needed for the study.

### Sampling Procedure

Multistage sampling method was used to randomly select six from 40 rural Kebeles in the Boset district. To identify eligible, the household census and numbering was carried out prior to data collection. Based on the identified households having the target group, the probability proportional to sample size allocation was carried out. Finally, systematic random sampling was used to include the eligible into the study. Indeed, only one was selected by lottery method if the household has two or more eligible to control for potential intra household correlation. For the Focus Group Discussions a minimum of two per each group was sampled and it was determined based on the saturation of information or redundancies

### Data Collection

Quantitative data were collected using structured interviewer administered questionnaire developed after reviewing relevant literatures^7^–^9^, ^11^. The questionnaire was translated into the regional working language (Afan Oromo) and translated back to English by experts (Supplementary File 1). Pretest of the questionnaire was carried out on similar setting on young females who had similar socio-demographic characteristics by considering 5% of the total sample size. The data were collected by 10th grade completed young females and supervised by Public Health officers. An interviewer paid a maximum of three visits to the selected households to locate respondents and in case of absenteeism eligible in the adjacent households has been interviewed. To ensure the validity of the data, 10% of the questionnaires were randomly checked for completeness by supervisors and principal investigator. A total of Eight FGDs was held among young people, elderly and religious leaders and governmental and Non-government employees. Discussions were facilitated using a discussion guide prepared according to the theme of the study. The discussions were audio recorded for later transcribed verbatim.

### Data Analysis

The collected data were cleaned, coded and entered into EpiInfo Version 3.1 and exported to SPSS 23 for analysis. Descriptive statistics were used to determine the proportion of sexual coercion among the study participants. The associations between the study variables were computed using binary logistic regression analysis. In the final model, variables having a P value of < 0.25 at bivariable level were chosen and entered into the final model using the forward selection method to estimate the adjusted effects of the predictor variables. The odds Ratio with 95% confidence intervals were calculated to indicate the strengths of the associations between the study variables. The significance of the association was declared at p-value of 0.05. The multicolliniarity between the independent variables using Variance Inflation Factor (VIF). Those variables having the value of 10 were excluded from the model. The model's ability to correctly classify the outcome was assessed using Receiver Operating Characteristic curve (ROC). For the qualitative information verbatim transcriptions were used. Texts were imported to Open Code 2007 program to facilitate the coding process^21^. Relevant cods were arranged into categories to arrive at predetermined themes and thematic analysis was conducted. The trustworthiness of qualitative information was ensured by recruiting appropriate discussants and member checking was also used after write up of the texts. The results of qualitative information were used to complement the quantitative results and discussions.

### Operational Definitions

Young people: are those who are in the age group of 10 to 24 years.

Sexual coercion: Unwanted or unwillingly completed penetration because of one or more of the followings: deception (promise) or reward, the threat of non-physical punishment or verbal pressure, exchange of sex for money/gifts/favor (transactional sex), by the use of physical force/rape and use of substances (alcohol, chat and drugs)^11^.

Sexual debut: Those young females experienced penetrative sexual intercourse for the first time^11^.

## Results

### Socio-demographic and behavioral Characteristics

It was planned to include 662 young females to the study and 632 were participated, making a response rate of 95%. One hundred twenty four (19.6%) were in the age range of 10- 14 years. The mean (+SD) age of the respondents was 17.2 (+3.3) years. Most (83.7%) were not married (single), while 13.8% were ever married. About seven in ten (70.4) of them were students and 4% of them were never attended education and considered to be illiterate during the data collection time. More than four in ten (41.9%) currently living with their parents, while 5.1% were living alone. About one of seven (14.7%) of the participants used alcoholic beverage and 2.7% chewed Khat (Table 1).

**Table 1:** Socio-demographic and behavioral Characteristics of the study participants, Boset district, June 2019

Variables	Responses category	Number	Percent
Age in years	10–14 15–19 20–24	124 332 176	19.6 52.5 27.9
Marital status	Unmarried Married Others*	530 87 15	83.7 13.8 2.5
Current occupation	Students Housewife Trade activity Gov. employee Housemaid Others***	470 66 21 6 42 27	74.4 10.4 3.3 0.9 6.6 4.4
Grade level	Illiterate Read and write Primary (1–8) Secondary (9–10) Preparatory (11–12) Collage and above	28 8 277 232 64 22	4.4 1.4 43.8 36.7 10.2 3.5
Currently living with	Father and Mother Father only/ only Mother Relatives Fiancé Spouse Friends Alone Maidservant	265 136 92 9 66 6 32 26	41.9 21.5 14.6 1.4 10.4 1.0 5.1 4.1
Consume Alcoholic beverage	Yes No	93 539	14.7 85.3
Chew Khat	Yes No	17 615	2.7 97.3

NB. Others include: *Divorced, widowed and separated, **Employed in private business, had no work

### Prevalence of Sexual Coercion at first Sexual debut

**Figure 1:**
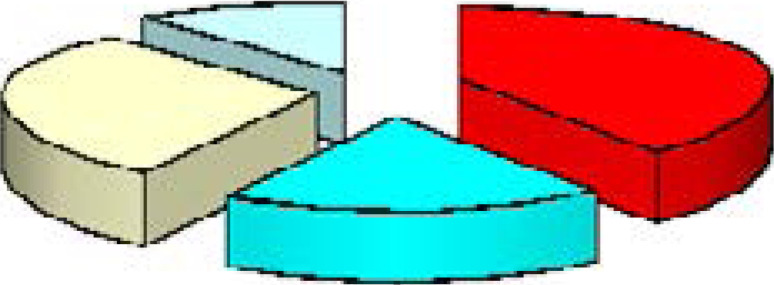
Reasons for sexual commencement of sexually active respondents, Boset district, Oromia Region, June 2019

The proportion of young female ever experienced penetrative sexual intercourse was 233 (36.9%). The mean (+SD) age at first sexual commencement was 16.64 (+1.97) years. The majority, 191 (81.8%) started to have sexual intercourse between the ages of 15–19 years, while 27 (11.7%) started between 10–14 years and only 15 (6.5%) were started between 20–24 years to have their first sex. From the reasons on sexual commencement for the first time, 85 (36.5%; 95%CI: 34.6%–38.4%)) mentioned due to sexual coercion, while because of marriage, falling in love and personal desire accounted for 59 (25.3%), 74 (31.7) and 15 (6.6%), respectively (Figure 1).

**Figure 2:**
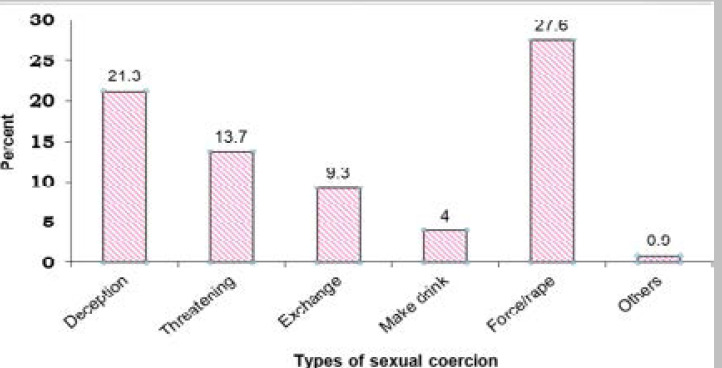
Types of Sexual coercion at first sexual debut among sexually active respondents, Boset district, Oromia Region, June 2019

Regarding the types of sexual coercion at first sexual debut, the victims faced multiple forms at the same time. More than a quarter (27.6%) were physically forced/raped, while nearly one in five (21.3) deceived or misled by promising words and the rest 13.7%, 9.3%, 4% and 0.9% of the victims started their first sexual intercourse by verbal threats, exchange for gifts/ property, by making them drunk or after chewing Khat and watching pornographic movies/pictures, respectively (Figure 2).

The result is substantiated by the results of FGD explored usually the sexual intercourse with girls is being practiced at the study area on the basis of economic support, physical forces/rape, and deception or misleading words coming from men for deflowering by repeatedly saying, “*I love you very much*”.

One of the discussant said “*Qalbiin debate amma ija xaafii geese, isseeyyuu yoo dhungatani lafa keessii*” meaning the discerning range of mind of females towards sexuality is as small as a seed of “millet”, which even she can lose it if kissed (38 years married women). This implies that females are easily flattered over sexual desire.

The discussants also mentioned pressure from peers is not uncommon. Men can make an agreement with their intimate friend of female that makes a mediating task in persuading another female (girls) for her friend so as to adjoin the unintended female with the man behind the trap is another system of sexual offenses or fornications in the area.

They further mentioned threatening girl friends to have sexual intercourse is another problem of sexual offense by saying, “I will find another girl, if you didn't allow me”, though she tries to convince him to wait until she completes her schooling. In line with this, men try to provoke girl friends to make sex by saying“*let me check whether you are virgin or not*”.

Literally they are using the local language “*Anoo durbummaakee hinballeessu nan borrosha malee*” meaning “*I don't deflower you, but to check it*.” (31 years male governmental employee)

In the same notion, the perpetrators used physical force to have sexual intercourse with young females. This was complemented by the results from FGDs and one young woman described the fact that:

“*Culturally, females are forced into sexual intercourse “Dubartiif harreen ulee jaallattii jechudhaan dubartii reebanii gudeedu*”. (23 years married women). Meaning, men make sexual intercourse with females by beating them even if they refuse, saying “*...females and donkey like beating by stick*”.

Other discussants from religious organizations recounting her first sexual experience with her boyfriend at age 15 said:

“*It was a terrifying experience; when I tried to resist, he pinned my arms above my head. It must have been so painful and suffocating that I fainted*”. (38 year married woman) (Supplementary file 2)

Moreover, most discussants mentioned, making females drink alcoholic beverages and Khat chewing for arousing her sexual feeling is a common mechanism for males to initiate females. According to this belief, forcing females into sexual intercourse after taking alcohol is a common practice by men even within a marital union.

The study showed for the relationship of perpetrators to victims, from the victims 23.8%, 27.7% and 18.8% were victimized by acquaintance, boyfriends and by their fiancées, respectively, whereas stranger victimized only 8.9%. According to the results from FGDs participants revealed the fact that some authority personnel's are also the perpetrators of sexual coercion by deceiving young females for employing into government work in exchange of sex. Other discussants claimed, teachers are also intimidating young females for passing examinations as well as giving favors and property, while some said; elderly people, married men, health professionals, acquaintance, passengers (casual), relatives, rich persons (business men), relatives, unemployed, drivers, gangs, etc. are the committers of sexual coercion. In addition, some of them mentioned, film watchers and alcohol initiated individuals are continually forcing females in to having sexual intercourse.

The estimated age of perpetrators during the time of sexual coercion was further asked and for one fourth (25.7%) of them it was about 1–4 years older than the victims, while 21.8% were 5–10 years older. Similarly, 71.3% of the perpetrators were single or never married and 13.9% were ever married when they were committed a sexual coercion the young females. For those who coerced at sexual debut, most (33.7%) of the events were occurring in the offender's house and dating place, including the in the bush. In doing so, 10.9% of the victims of sexual coercion have reported the events to legal bodies, their families and friends. Among those reasons mentioned for not reporting, fear of consequences from the act (58.9%), fear of social stigma from the community (46.7%), cultural influences (30%) and the threat of harm by the offender (41.1%) were mentioned. Moreover, few (16.8%) of all cases sought health institutions for help (Table 2).

**Table 2:** Characteristics of perpetrators of sexual coercion at sexual debut among sexually active respondents, Boset district, Oromia Region, June 2019

Variables	Response category	Frequency	Percent
Relation of perpetrators to victims (n= 85)	An acquaintance A friend Fiancé Spouse A relative Teachers Authority figures Strangers	20 24 16 7 3 7 1 8	23.8 27.7 18.8 7.9 4.0 7.9 1.0 8.9
Estimated age of perpetrators (n= 85)	About her age Younger than her About 1 to 4 years older About 5 to 10years older More than 10 years older Don't know	14 16 22 19 5 9	16.8 18.8 25.7 21.8 6.0 10.9
Place of incident happened (n= 85)	At victims' home Neighbor At school Hotel On street during the night At the perpetrator's house, dating	14 11 7 20 5 29	16.8 12.9 6.6 23.8 5.9 33.7
Reported the incident (n= 85)	Yes No	9 76	10.9 89.1
Place to report (n= 9) *(The percentage may not* *add to 100, as multiple* *responses are possible)*	Family Friends Relatives Legal bodies/Police, Judge Health providers	2 6 2 4 2	18.2 63.6 18.2 45.5 27.5
Reasons for not to have report (n= 76) *(The percentage may not* *add to 100, as multiple* *responses are possible)*	Fear of social stigma Cultural influence Fear of consequences from family Threat of harm by perpetrator Fear of friends	35 23 45 31 3	46.7 30.0 58.9 41.1 4.3
Seek health institutions for help (n= 85)	Yes No	14 71	16.8 83.2

Factors associated with Sexual Coercion at sexual debut Those married young females and living with their spouse reported less likelihood of sexual coercion at sexual debut compared to never married young females (AOR, 0.71; 95%CI: 42,0.81). But, it was no more significant after being adjusted all other factors. On the other hand, considering young females living with their parents, those living alone had significantly increased likelihood of having sexual coercion at sexual debut (AOR, 5.07; 95%CI: 1.07,24.01). Also, age at first sexual commencement of young females was found to be a significant predictor, with young female 15–19years were significantly less likely to report having experienced sexual coercion at sexual debut compared to 10–14 years (AOR, 0.19; 95%CI: 0. 06, 0.54). Ever consuming alcoholic beverages by young females was significantly associated with sexual coercion at sexual debut (AOR, 2.44; 95%CI: 1.17, 5.08). Similarly, those young females who had ever chewed khat had a significantly higher likelihood of having sexual coercion at sexual debut (AOR, 8.30; 95%CI: 1.89, 36.38) (Table 3).

**Table 3:** Factors associated with sexual coercion at sexual debut among respondents, Boset district, Oromia Region, June 2019

Variables	Sexual coercion at first sexual debut		COR (95% CI)	AOR (95% CI)
	Yes-%	No- %		
**Marital status** Never married Married Others*	67(48.6) 21(26.9) 13(72.3)	71(51.4) 57(73.1) 5(27.3)	1.00 0.39 (0.21,0.71)* 2.76 (0.93,8.95)	1.00 0.71 (0.42,0.81)* 0.83 (0.15,4.69)
**Living arrangements** (Currently live with) Both parents Father/Mother only Relatives, Fiancé or friend Spouse Alone Housemaid	23(46.0) 22(46.8) 16(44.4) 15(22.7) 14(70.0) 11(78.6)	27(54.0) 25(53.2) 20(55.6) 51(77.3) 6(30.0) 4 (21.4)	1.00 1.03 (0.47, 2.23) 0.94 (0.40,2.22) 0.35 (0.16,0.77)* 2.74 (0.91,8.28) 4.30 (1.07,17.32*	1.00 1.27 (0.47,3.44) 1.01 (0.34,2.99) 0.53 (0.11,2.51) 5.07 (1.07,24.01)* 3.49 (0.49,24.59)
**Current occupation** Student Housewife Trade activity House maid Gov. employee /Others	42(42.4) 17(25.4) 13(56.5) 19(67.9) 10(58.8)	57(57.6) 50(74.6) 10(43.5) 9(32.1) 7(41.2)	1.00 0.461(0.23,0.91)* 1.76 (0.71,4.41) 2.87 (1.18,6.66)* 1.93 (0.68,5.51)	1.00 0.77 (0.23,2.62) 1.56 (0.38,6.31) 2.40 (0.79,7.34) 2.90 (0.83,10.18)
**Age at first sex** 10–14 years 15–19 years 20–24 years	17(63.0) 75(40.3) 5(33.3)	10(37.0) 101(59.7) 10(66.7)	1.00 0.40(0.17,0.92)* 0.29(0.08,1.11)	1.00 0.17 (0.06,0.54)* 0.33 (0.06,1.83)
**Consume alcoholic beverage** No Yes	58(36.7) 43(56.6)	100(63.3) 33(43.4)	1.00 2.25(1.29, 3.92)*	1.00 2.44 (1.167,5.08)*
**Chew chat** No Yes	86(39.8) 15(83.3)	130(60.2) 3(16.7)	1.00 7.56(2.12,26.89)*	1.00 8.30 (1.89,36.38)*

**Adjusted for the variables in the table**, * P- Value <0.05

COR- Crude Odds Ratio, AOR- Adjusted Odds Ratio

Note: variables not entered in the model because they were not found significant in bivariate analysis. Others include: *Divorced, widowed and separated.

## Discussion

This study used a community based cross-sectional design using a mixed method of data collection method and highlighted the prevalence of sexual coercion at sexual debut and associated factors among young females in rural Ethiopia.

From the sexually experienced young females the reported reasons for sexual commencement, sexual coercion is the highest and constituted 36.5%. This finding is consistent with the reports from different studies around the African countries indicated the prevalence of nonconsensual first sexual experiences among young female of 33.1% in Uganda^22^ and 27.5% in Nigeria^15^. Similar reports were shown in a study among young females in University and College students of Ethiopia that showed 43.3% in Bishoftu town^23^, and 45.4% in Wolayita Sodo^18^. The variation in prevalence is attributed to the differences in the study settings in which the majority of the studies were conducted in school and urban settings. The majority of FGD discussants approved for these reasons as a cause of sexual debut for young females and further mentioned the issues considering as a serious problem of the study area.

In this study, the greater proportion (23.4%) of sexually coerced young females at sexual debut reported sex by the use of physical force/rape. This is more than 12.7% in Addis Ababa and 20.8% from Nekemte town^24^. These might be due to urban rural disparities as rape is more common in rural communities of Ethiopia as cultural acceptance is evidenced from the results of FGDs. For a significant proportion of the victims, boyfriends (27.7%) and acquaintances (23.8%) were the perpetrators of sexual coercion at sexual debut and a smaller proportion (8.9%) were coerced by an unknown person to the victim (strangers). It is comparable with similar reports from Kenya, which indicated that acquaintances and strangers perpetrated 21.8% and 8.2% of sexual coercions at sexual debut, respectively^25^. With regard to the age of the perpetrators, only 16.8% of the victims were reported about the same age, while the majority reported for the age of the perpetrators was more than the victim's age, even 5.9% of them were reported to about 10 years and above differences. This shows that some young female experience non-consensual sexual relationships with older, more powerful partners with whom they may feel unable to negotiate and perform unsafe sexual practices.

Married young women were less likely than single to report sexual coercion at sexual debut. This is different from similar studies in Kenya, which showed young females who had ever been married, and those who did not live with their parents had a significantly elevated risk of sexual coercion at sexual debut^25^. This is corroborating the notion that marriage is a safe refuge especially for the young females. Young females living alone were significantly at higher risk of getting sexual coercion al debut than those living with their parents. Similar findings were reported in other similar studies^14^, ^24^, ^26^. This was complemented by most discussants from FGDs mentioning for the young females living away from parents are more vulnerable to sexual coercion. The study also showed a significant association between earlier onset of sexual relations among young females and greater risk of sexual coercion. This is supported by the reports from studies conducted elsewhere, explaining for being earlier age is vulnerable to unwanted sexual intercourse and young female who initiated sex at very young ages may also have experienced some sort of pressure either physical or verbal to have sex against their will^4^, ^6^, ^22^, ^25^.

This study highlights the prominent role of drinking alcoholic beverages and chewing khat by young female increasing the risk of sexual coercion at sexual debut. These are evidenced by various studies on the subject area^4^, ^17^, ^23^, ^24^. The plausible explanation for these might be behavioral risk factors that put young females at increased vulnerability to sexual coercion, because they might not control themselves over sexual feelings by interpreting and effectively acting on warning signs. All FGDs group briefly mentioned the role of alcohol and Khat use in association with sexual coercion for the young female in the study area.

As to the limitation of this study, due to the cores sectional nature it might not be possible to ascertain the direction of the cause effect relationships between the study variables. Also, social desirability bias was inevitable due to the sensitive nature of the study. Despite these limitations it is possible to generalize the findings of this study to similar rural settings having similar socio-demographic characteristics in the country.

## Conclusion

Sexual coercion at sexual debut constitutes the largest proportion among other reasons for sexual commencement. Sexual experiences using physical force, Rape by the perpetrator constitutes the highest proportion from the defined forms of sexual coercion at sexual debut. The perpetrators of sexual coercion at sexual debut were mostly boyfriends and acquaintances.

Those unmarried young females and living alone (away from their parents) were at increased likelihood of sexual coercion at sexual debut. Similarly, young female who started sexual intercourse earlier, and drinking alcoholic beverages as well as chewing khat were significantly at increased risk of having sexual coercion at sexual debut. The emerging program interventions need to take into consideration in order to address the full context of young female lives, including the societal and behavioral factors leading to these problems. Moreover, there is a need to improve the communication skill and assertiveness through life skill training for young females in rural areas.

## Abbreviations

AOR: Adjusted Odds Ratio
COR: Crude Odds Ratio
IRB: Institutional Review Board
SD: Standard Deviation

## Declarations

### Availability of data and materials

All data generated or analyzed during this study are included

## Author details

The Author is a public Health specialist and associate professor of public health. Currently teaching postgraduate students in the Adama Hospital Medical College and advising the Federal Ministry of Health of Ethiopia on the areas of Reproductive, Maternal, Neonatal, Child and adolescent health.
